# The involvement of osteopontin and matrix metalloproteinase- 9 in the migration of endometrial epithelial cells in patients with endometriosis

**DOI:** 10.1186/s12958-015-0090-4

**Published:** 2015-08-20

**Authors:** Mei Yang, Chunfan Jiang, Hua Chen, Yan Nian, Zhimiao Bai, Chunfang Ha

**Affiliations:** Ningxia Medical University, Yinchuan, Ningxia China; Department of Obstetrics and Gynecology, Xiangyang Central Hospital, Affiliated Hospital of Hubei University of Arts and Science, Xiangyang, Hubei China; Department of Pathology, Xiangyang Central Hospital, Affiliated Hospital of Hubei University of Arts and Science, Xiangyang, Hubei China; Department of Obstetrics and Gynecology in General Hospital, Key Laboratory of Fertility Preservation and Maintenance of the Ministry of Education, Ningxia Medical University, Yinchuan, Ningxia China

**Keywords:** Endometriosis, Osteopontin, Estrogen, Matrix metalloproteinase-9, Migration

## Abstract

**Background:**

Endometriosis, which shares certain characteristics with cancers, may cause abnormal expression of proteins involved in cell migration. Endometrial epithelial cells (EECs) are believed to play an important role in endometriotic migration. The aim of this study was to investigate the relationship between the expression of osteopontin (OPN) and matrix metalloproteinase-9 (MMP-9) in endometriotic migration.

**Methods:**

We performed primary culture of EECs and investigated the expression of OPN and MMP-9 in EECs regulated by 17beta-estradiol (E2). OPN-specific siRNA interference was used to down-regulate OPN and to explore the corresponding change in MMP-9 expression. Real-time RT-PCR, western blot analysis and flow cytometry were used to determine the expression levels of OPN and MMP-9. Gelatin zymography was performed to observe the enzymatic activity of MMP-9 in conditioned media. Transwell and wound scratch assays were performed to investigate the migration ability of EECs.

**Results:**

The expression levels of OPN and MMP-9 in normal EECs (NEECs) were inferior to those in EECs from patients with endometriosis (EEECs). The expression levels of OPN and MMP-9 from stage III/IV EEECs and secretory-phase EECs were higher than those of stage I/II EEECs or proliferative-phase EECs. The expression levels of OPN and MMP-9 in EEECs were increased by E2 treatment and remarkably decreased by siRNA interference. Active MMP-9 expression increased with E2 treatment and decreased with siRNA treatment in EEECs compared with the same treatments in NEECs. The migratory abilities of EEECs were enhanced after cells were treated with E2; in contrast, these abilities were reduced by siRNA interference. In NEECs, active MMP-9 and cellular migration abilities were only minimally influenced by E2 and siRNA treatment.

**Conclusions:**

The present study suggests that the up-regulation of MMP-9 via activation of OPN induced by estrogen may correlate with the migration of endometrial epithelial cells in patients with endometriosis.

## Introduction

Endometriosis is a common chronic gynecological disease affecting 10 % of women of child-bearing age. Symptoms include dysmenorrhea, chronic pelvic pain, menorrhagia and infertility. Individual susceptibility to endometriosis includes a complex interaction of genetic, hormonal and environmental factors [1]. Although several theories, such as retrograde menstruation, coelomic metaplasia and Müllerianosis, have been proposed to explain its etiology, the molecular mechanisms of this disease remain unclear.

Endometriosis can cause endometrial cells to invade and metastasize to various organs and even compromise the functions of these organs. In a sense, endometriosis shares some similar characteristics with cancers [2]. Many proteins have been found to play roles in the invasion and metastasis of cancer cells; however, whether these proteins exert a similar influence on endometrial cells in endometriosis has not yet been fully investigated.

Osteopontin (OPN) is an integrin-binding glycoprotein that was first discovered in the bone matrix and then implicated in adhesion, anti-apoptosis and metastasis in cancer cells [3, 4]. Matrix metalloproteinase-9 (MMP-9), also known as the largest gelatinase in the family of matrix metalloproteinases, is a zinc-dependent endopeptidase that facilitates the degradation of type IV collagen in many cancers. Recently, it was reported that OPN activates the PI3-K/Akt pathway, up-regulates HIF-1alpha via binding to v3 integrins and promotes the degradation of the extracellular matrix through uPA and MMP-9 to mediate cancer cell metastasis [5]. Another study has reported that OPN promotes the progression of gastric cancer by activating matrix metalloproteinase 2 (MMP-2) and MMP-9 through the NF-kappaB pathway [6]. The OPN/MMP-9 pathway represents a new molecular mechanism involved in cancer metastasis.

OPN and MMP-9 expression levels have been determined in normal endometrial epithelial cells (NEECs). A much stronger OPN expression is found in the middle and late secretory phases compared with the proliferative and early secretory phases [7, 8]. MMP-9 is much more strongly expressed in the late secretory phase than in the other phases of the menstrual cycle [9, 10]. The elevated expression levels of OPN and MMP-9 in the coincident phase of the menstrual cycle suggest that there may be a correlation between the expressions of these two proteins. Various studies on OPN expression in patients with endometriosis have provided similar results. OPN mRNA and protein expression levels, as well as levels of the secreted glycoprotein in the blood, are remarkably increased in endometriotic lesions compared with normal tissues [11, 12]. MMP-9 plays an important role in the changes in the growing cycle and tissue breakdown that occur in the endometrium and affects the migration of endometrial cells in endometriotic lesions. Several recent studies have examined the increased expression of MMP-9 in patients with endometriosis [13, 14]. Thus, we hypothesize that a signal-transduction pathway involving OPN and MMP-9, similar to that in cancer cells, may play a role in the migration of endometrial epithelial cells (EECs) in endometriotic lesions.

Currently, endometriosis is regarded as an estrogen-dependent disorder, and the initiation and development of this disorder can be promoted by systemic or locally synthesized estrogen [15]. The local concentration of estrogen is much more extensively regulated by intra-tissue estrogen metabolism than in the serum [16]. Gonadotropin-releasing hormone agonists (GnRH-a) and aromatase inhibitors are widely used to treat endometriosis [17, 18]. These agents inhibit the proliferation and metastasis of endometrial cells and accelerate the apoptosis of these cells by inducing a low local estrogen level [19, 20]. Therefore, estrogen at the local level may be associated with the metastasis and proliferation of endometrial cells in endometriosis. Currently, the pathological mechanisms by which OPN and MMP-9 levels are affected by estrogen are not fully elucidated and require further study. Based on the findings above, we hypothesize that OPN might up-regulate MMP-9 expression as a result of the locally increased concentration of estrogen, which promotes the migration of endometrial cells and eventually leads to endometriosis.

The present study was designed to explore the regulatory correlation between OPN and MMP-9 in EECs. We performed primary cell culture to acquire highly pure EECs. Estrogen (17beta-estradiol, E2) was used to influence OPN and MMP-9 expression in EECs. We examined expression of MMP-9 and the migratory ability of EECs after treatment with an OPN-specific siRNA and E2. Real-time RT-PCR, western blots and flow cytometry were used to detect the expression levels of OPN and MMP-9. Gelatin zymography was performed to observe the enzymatic activity of MMP-9 in conditioned media. Transwell and wound scratch assays were conducted to determine the migratory ability of EECs.

## Methods

### Ethical approval

This study was carried out in accordance with the Declaration of Helsinki. Ethical approval was given by the Ningxia Medical University Institutional Review Board (reference number 20130106) on October 4^th^, 2013. Written informed consent was obtained from all of the recruited patients. This study was initiated on November 13^th^, 2013 and terminated on August 20^th^, 2014.

### Patient recruitment and characterization

AII of the patients recruited in this study were women of child-bearing age. Thirty-two women with endometriosis who had not received hormones or GnRH-a agonist treatment for at least six months were recruited before surgery, and all underwent laparoscopic surgery at the Department of Obstetrics and Gynecology of General Hospital, Ningxia Medical University, Ningxia, China. Among these 32 patients, preoperative diagnosis included 18 with an ovarian cyst and 14 with infertility. Each case of endometriosis was staged during the operation according to the revised American Fertility Society classification of endometriosis (rAFS) and subsequently confirmed by histology. Thirty women undergoing tubal ligation for sterilization were recruited as controls. No minimal endometriosis was found in these control subjects. The endometrium from patients with endometriosis was obtained by Pipelle biopsy during diagnostic laparoscopy or by uterine curettage for the control subjects. Detailed information on the study participants is listed in Table 1.

**Table 1:** Clinical characteristics of patients

	Control	Endometriosis
Age (mean ± SD)	30.5 ± 1.1	32.4 ± 4.2
Age range (yr)	27-35	21-38
rAFS staging: I–II/III–IV		10/22
Menstrual cycle: P/S	18/12	19/13

P, proliferative phase; S, secretory phase

### Primary culture of endometrial epithelial cells

All tissues were washed three times with sterile Hank’s Balanced Salt Solution (HBSS, phenol red free), minced into pieces of approximately 1 mm^3^ and digested in 10 ml of HBSS containing type IV collagenase (0.03 %; Sigma, St. Louis, MO) and 10 U/ml DNase I (Sigma) at 37 °C for 40 min. Type IV collagenase is an enzyme that is believed to degrade cellular mesenchyme such as laminin, type IV collagen and basal membrane while preserving the integrity of epithelial cells. Next, the undigested tissues were collected by centrifugation at 500 × g for 1 min, and the sediment was subjected to another round of digestion for 40 min. Epithelial cells and stromal cells in the supernatant were separated by differential centrifugation [21]. Selective attachment was carried out to repurify the endometrial cells [22]. The endometrial cells were cultured in phenol-red-free DMEM/Ham’s F12 (Invitrogen, Carlsbad, CA) supplemented with 10 % v/v fetal bovine serum (FBS; Invitrogen), 100 μg/ml streptomycin, 100 U/ml penicillin and 2 μg/ml of Fungizone to confluence. Next, they were subjected to differential trypsinization and attachment for further purification. They were then washed in phosphate-buffered saline (PBS; pH = 7.4) and selectively trypsinized with a 1:6 dilution of 1× trypsin/EDTA (Sigma) until the contaminating stromal cells were removed. After washing the cells in PBS, the trypsin/EDTA solution was removed, and epithelial cells were plated (2 × 10^4^ cells/ml) in dishes in DMEM/Ham’s F12 supplemented with 10 % charcoal-dextran stripped FBS (Invitrogen).

### Immunocytochemistry

The procedure used for immunocytochemical staining has been described previously [23]. Briefly, EECs were cultured on a sterile cover glass in six-well plates. When 70 % confluence was reached, cells were fixed with 4 % paraformaldehyde at room temperature for 30 min. Vimentin (Vim), pan CK, OPN and MMP-9 (Abcam for all) were used as the primary antibodies. The dilution ratios were as follows: vimentin (1:500), pan CK (1:500), OPN (1:200) and MMP-9 (1:300). Next, cells were incubated with a secondary antibody (PV9000; Zymed Laboratory, South San Francisco) and visualized with 3, 3’-diaminobenzidine tetrahydrochloride (DAB; Golden Bridge, Beijing, China). Cells were then subjected to nuclear counterstaining (blue staining) with hematoxylin.

### Drug intervention

When EECs reached 60 % confluence, the media was discarded and the cells were cultured in DMEM/Ham’s F12 without FBS for 12 h. Next, the media were changed to DMEM/Ham’s F12 plus 10 % charcoal-dextran stripped FBS supplemented with E2 at 0, 0.1, 0.5, 1, 5 or 10 nM, and EECs were cultured for 48 h. The mRNA levels of OPN and MMP-9 reached their maximum at a specific E2 concentration called the optimum concentration. EECs were again cultured according to the protocols described above. We treated EECs with the optimum concentration of E2 for 0, 12, 24, 36, 48, 60 or 72 h. At each time point, the mRNA levels of OPN and MMP-9 were determined by real-time RT-PCR. The time point at which the mRNAs of OPN and MMP-9 peaked was referred to as the optimum time point. In the subsequent experiments, including flow cytometry, gene silencing, western blots and gelatin zymography, EECs were treated with E2 at the optimum concentration and time point. Each experiment was performed three times, and each group was repeated in triplicate.

### Flow cytometry

After EECs were treated with E2 for 48 h (the optimum time point) to allow for adequate protein synthesis, fluorescently tagged mouse-anti-human MMP-9 antibodies (BD Biosciences) wasused to detect antigens on the cells according to the following procedure. Approximately 1 × 10^6^ cells were fixed with PBS containing 4 % paraformaldehyde for 30 min. After the EECs were washed in PBS three times, they were suspended in PBS supplemented with 0.5 % bovine serum albumin (BSA). EECs were blocked with 1 mg of mouse IgG (Beyotime Biotechnology, Jiangsu, China) for 15 min and then incubated with the OPN and MMP-9 antibodies at room temperature for 1 h. Unbound antibodies were removed by washing with PBS, and cells were again resuspended in PBS. EECs were analyzed on a FACSAria II Flow Cytometer (BD Biosciences, San Jose, USA). EECs not stained with antibodies were used to define gating. EECs not treated with E2 were used as controls.

### Gene silencing

EECs were divided into two groups (siRNA-OPN and siRNA-scrambled). Scrambled siRNA was used as a negative control. The OPN siRNA sequences were as follows: sense, 5′ -GGUCAAAAUCUAAGAAGUUTT-3′ and antisense, 5′-AACUUCUUAGAUUUUGACCTC-3′; the scrambled siRNA sequences were as follows: sense, 5′-UUCUCCGAACGUGUCACGUTT-3′ and antisense, 5′-ACGUGACACGUUCGGAGAATT-3′. All of the siRNAs were designed and purchased from Shanghai GenePharma Inc. The siRNA oligonucleotides were transfected into EECs using X-tremeGENE reagent (Roche Diagnostics). The ratio of transfection reagent to siRNA was 10 μl: 2.5 μg per 2 × 10^5^ cells. The transfection procedure was carried out according to the manufacturer's instructions. The efficiency of siRNA interference was examined by western blot 72 h later.

### RNA extraction and real-time RT-PCR

Trizol (Invitrogen), chloroform, isopropanol and ethanol were successively used to extract the total RNA from the EECs. RNA concentration was assessed using a NanoDrop spectrophotometer (Thermo Scientific, Waltham, MA). For each sample, 1 μg of total RNA was used to synthesize cDNA with a TIANscript reverse transcription kit (TIANGEN Biotech, Beijing, China) according to the manufacturer’s instructions. Real-time RT-PCR was performed in triplicate for each sample using SYBR Premix Ex Taq II (TaKaRa Biotech, Dalian, China) according to the manufacturer’s instructions. Reactions were carried out using a Roche LightCycler480. Amplification of beta-actin was used for normalization. The primers used in this assay were as follows: OPN: sense 5’- ACAGCCGTGGGAAGGACAGTTA -3’, antisense, 5’- CCTGACTATCAATCACATCGGAATG -3’; MMP-9: sense, 5’-AGTCCACCCTTGTGCTCTTCCC -3’, antisense, 5’-TCTGCCACCCGAGTGTAACCAT-3’; beta-actin: sense, 5’-AGCGAGCATCCCCCAAAGTT-3’, antisense, 5’-GGGCACGAAGGCTCATCATT-3’. The relative expression levels of each gene were determined with the 2^−ΔΔCt^ method [24].

### Western blot

A total of 1 × 10^7^ EECs was washed twice with cold PBS and then harvested in 250 μl RIPA lysis buffer with 10 μl protease inhibitor cocktail (Sigma-Aldrich). Protein aliquots (50 μg) were run on a 10 % SDS polyacrylamide gel and transferred to a nitrocellulose membrane (Amersham). The membrane was blocked in 5 % milk in PBS containing 0.1 % Tween 20 (PBS-T) at room temperature for 1 h. The membrane was incubated with 1 % milk in PBS-T containing a mouse anti-human monoclonal OPN (1:1,000, Abcam) or MMP-9 (1:500, Abcam) antibody at 4 °C overnight. A rabbit anti-human polyclonal beta-actin antibody (1:2,000; Beyotime) was used as the internal control. The membrane was then probed with goat anti-mouse or anti-rabbit IgG conjugated to horseradish peroxidase (1:5,000 for both; Beyotime) in 1 % milk in PBS-T for 1 h. Immunoreactive proteins were detected with an enhanced chemiluminescence (ECL) reagent (Amersham) and exposed to X-ray film (FUJIFILM, Japan). The optical density of each band was analyzed with ImageJ (http://rsb.info.nih.gov/ij/download.html).

### Gelatin zymography

EECs were treated with siRNA or E2 according to the procedures described above. For the siRNA-OPN+ E2 group, EECs were treated with siRNA-OPN and E2 together with the same concentrations used in the separate drug treatment described above for 48 h. The enzymatic activity of MMP-9 in the media was detected with gelatin zymography as described previously with minor modifications [25]. Briefly, the conditioned media were subjected to 10 % polyacrylamide gel electrophoresis in gels containing 1 mg/ml gelatin. Next, the gels were washed with 2.5 % Triton X-100 (v:v) and 50 mM Tris–HCl (pH 7.5) at room temperature for 2 h. After that, the gels were incubated in a buffer supplemented with 10 mM CaCl_2_, 150 mM NaCl and 50 mM Tris–HCl (pH 7.5). The gels were then stained with 0.25 % (w/v) Coomassie blue R-250 in 30 % methanol and 10 % acetic acid for 30 min at room temperature and then de-stained with a solution containing 50 % methanol and 10 % acetic acid to show areas with gelatinase activity. The gels were photographed with Bio-Rad Gel Doc XR+, and the gray density of the bands was analyzed using Image Lab™ Software, Version 3.0.

### Transwell assay

One layer of 2 % Matrigel was used to cover the top chamber of 24-well micropore polycarbonate membrane inserts (8 μm; Millipore, Billerica, MA). EECs were treated with siRNA-OPN, E2 or siRNA-OPN + E2 as described above. Approximately 1 × 10^5^ trypsinized cells per well were seeded into the top chamber using FBS-free DMEM/Ham’s F12. DMEM/Ham’s F12 with 10 % charcoal-dextran stripped FBS was added to the lower chamber as a chemoattractant. The plate was placed at 37 °C for 24 h. Assays were then stopped by removing the non-invading cells in the top chamber with swabs. Cells on the lower surface of the membrane were fixed with 4 % paraformaldehyde and stained with hematoxylin. Cells in five visual fields per insert were counted and photographed (400× magnification).

### Wound scratch assay

EECs were evenly plated in a 12-well culture plate (1 × 10^6^ cells/well) and allowed to reach 90 % confluence. Wounds were made by scratching the cell layer using a 1000-μl sterile pipette tip along a ruler. Cellular debris was removed thoroughly by washing the scratched area repeatedly with PBS. In the presence of serum, untreated cells should migrate and fill the scratch area after approximately 72 h. Forty-eight hours after scratching, different treatments displayed remarkable effects on cellular migration in preliminary experiments, so this time point was chosen to end the assay. EECs were treated with siRNA-OPN, E2 or siRNA-OPN + E2 as described above. Images were acquired using an Olympus IX71 inverted microscope (100× magnification). The images shown are representative of three independent experiments. The area of unfilled wounds in five random fields was measured with ImageJ for further quantitative analysis. Wound area (%) = 48-h scratch wound/0-h scratch wound × 100 %.

### Statistics

All repeated data are shown as the mean ± SEM. A one-way ANOVA was used to compare the differences among NEECs and EEECs for various treatments. P values < 0.05 were considered statistically significant. SPSS 15.0 (SPSS Inc., Chicago, IL, USA) was used for all statistical analyses. PRISM 5.0 for Windows (GraphPad, San Diego, CA, USA) was used to create the histograms.

## Results

### Identification of the primary cultured cells

**Fig. 1:**
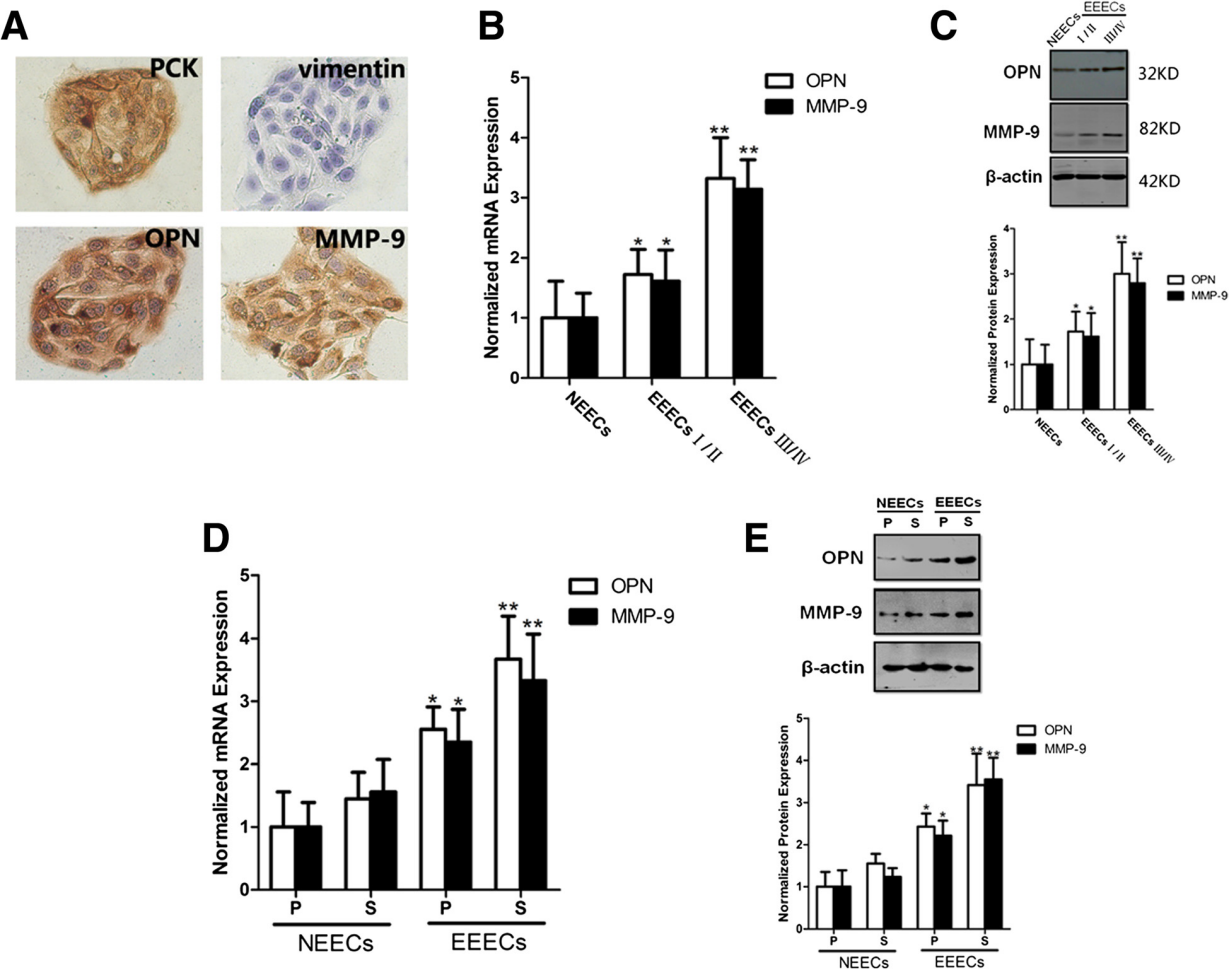
Vim, pan CK, OPN and MMP-9 were identified in EECs with immunocytochemistry. **a** The isolated EECs display distinct cobblestone morphology and express pan CK instead of Vim. OPN and MMP-9 are located in the cytoplasm of EECs. OPN and MMP-9 expression levels are detected in NEECs and EEECs with real-time RT-PCR (**b**) and western blots (**c**). *, I/II EEECs vs. NEECs, *p* < 0.05; **, III/IV EEECs vs. NEECs, *p* < 0.01. Real-time RT-PCR (**d**) and western blots (**e**) show that OPN and MMP-9 expression is detected in NEECs and EEECs. *, proliferative phase EEECs vs. proliferative phase NEECs, *p* < 0.05; **, secretory phase III/IV EEECs vs. secretory phase NEECs, *p* < 0.01. p, proliferative phase; s, secretory phase. The histogram represents three independent assays. Data are presented as the mean ± S.E. *The* housekeeping gene beta actin was used to normalize the expression levels of OPN and MMP-9

Using primary culture techniques, we successfully isolated EECs. EECs grew clonally in clumps and displayed a typical cobblestone morphology (Fig. 1a). To ensure that the cultured cells were endometrial epithelial cells, we identified different cell markers with immunocytochemistry. As expected, most cells were positive for pan CK (immunostaining in the cytoplasm) but negative for vimentin (Fig. 1a). The pan CK biomarker is characteristic of cells of epithelial origin, whereas vimentin labels cells to be of mesenchymal origin. The successful primary culture of endometrial epithelial cells laid the foundation for subsequent assays.

### Detection of OPN and MMP-9 expression in EECs by immunocytochemistry

We detected the expression levels of OPN and MMP-9 in the primary cultured EECs by immunocytochemistry. OPN immunostaining was located in the cytoplasm of most EECs, and the MMP-9 distribution pattern was similar to that of OPN (Fig. 1a).

### Quantification of the OPN and MMP-9 expression levels with real-time RT-PCR and western blots

To compare the quantitative differences in OPN and MMP-9 expression levels in NEECs and eutopic EECs with endometriosis (EEECs), we performed real-time RT-PCR and western blots. We used the 2^−ΔΔCt^ method to obtain the relative expression values of these two genes. To normalize the expression levels of OPN and MMP-9, the relative gene expression levels from NEECs were set to 1, and the normalized mRNA expression of OPN from stage I/II and stage III/IV EEECs were 1.59 and 3.32, while those of MMP-9 were 1.68 and 3.14, respectively (Fig. 1b). Statistical analysis showed that significant differences in the expression levels of OPN and MMP-9 existed between any two groups of NEECs, stage I/II and stage III/IV EEECs (*p* = 0.031). Similarly, the gray density ratio of OPN (MMP-9)/beta-actin was used to represent the relative expression levels of these two proteins. After normalization, the expressions of OPN from stage I/II and stage III/IV EEECs were 1.69 and 3.01, while those of MMP-9 were 1.57 and 2.79, respectively (Fig. 1c). There were significant differences in the expression levels of OPN and MMP-9 between any two groups of NEECs, stage I/II and stage III/IV EEECs (*p* = 0.028). The relative gene expression levels of OPN and MMP-9 in the proliferative phase from NEECs were set to 1. The normalized mRNA expressions of OPN and MMP-9 in the secretory phase of NEECs were 1.45 and 1.56. Similarly, the OPN mRNA expression in the proliferative phase and secretory phase of EEECs were 2.61 and 3.60, while that of MMP-9 was 2.74 and 3.31, respectively. Significant differences in the expression levels of OPN and MMP-9 were observed when comparing different stages of the menstrual cycle in both NEECs and EEECs (*p* = 0.025) and also in the same stage of the menstrual cycle when comparing NEECs and EEECs (*p* = 0.0031). We also compared the normalized protein expressions of OPN and MMP-9 in different menstrual cycle stages in NEECs and EEECs, and significant differences were found between different menstrual cycle stages in both NEECs and EEECs (*p* = 0.010) and between NEECs and EEECs in the same menstrual cycle stage (*p* = 0.0015). The expression levels of OPN and MMP-9 from NEECs were lower overall than those from EEECs.

### E2 induced OPN and MMP-9 expression

**Fig. 2:**
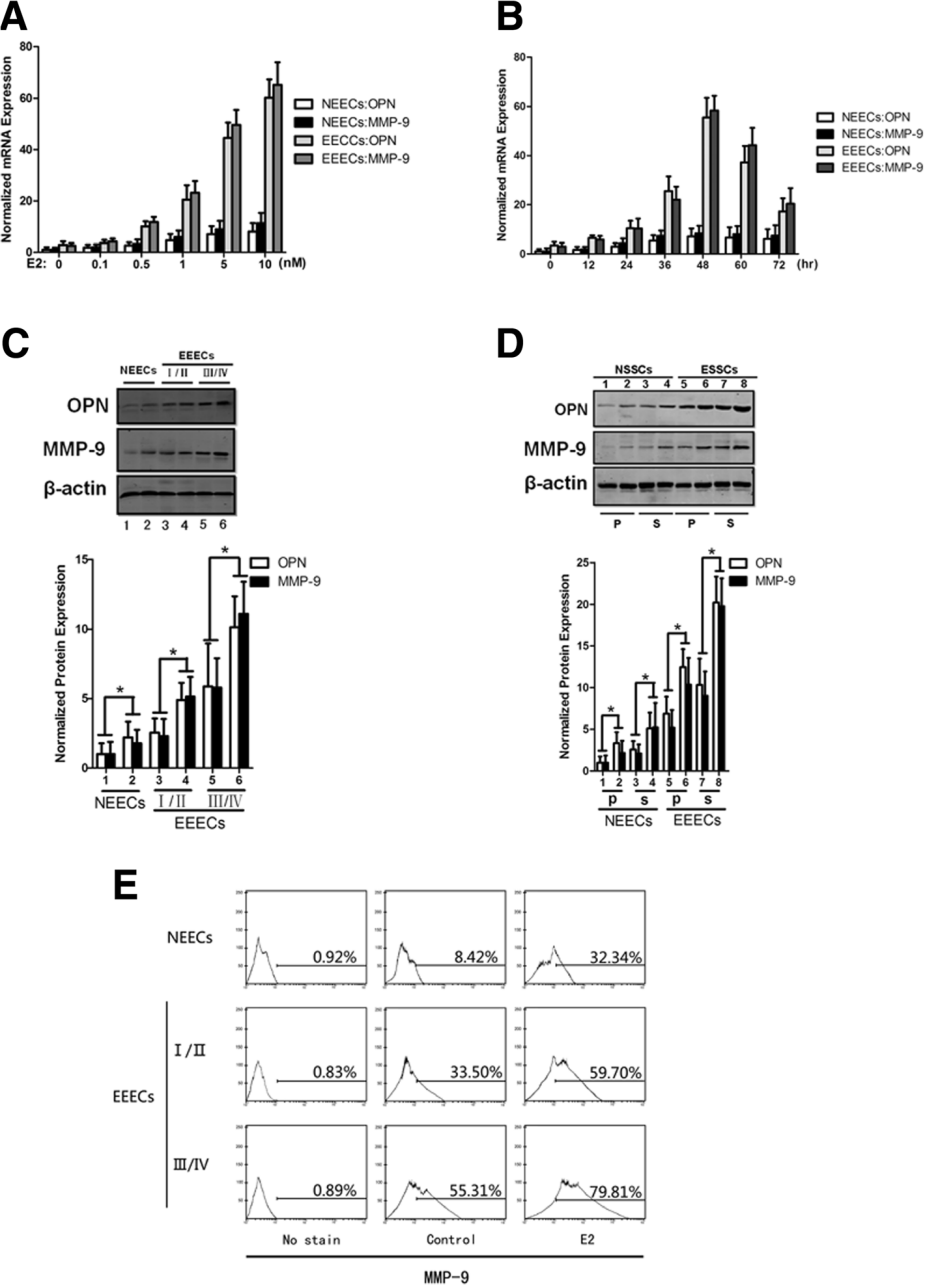
E2 induced OPN and MMP-9 expression in EECs. A dose–dependent response (**a**) and a time-dependent response (**b**) based on real-time RT-PCR depicts the changes in OPN and MMP-9 expression after stage III/IV EEECs were treated with E2. Typical bands of western blots and the histogram are shown for E2 treatment at the optimum concentration (10 nM) and time point (48 h) grouped by rAFS staging (**c**) or by menstrual cycle (**d**). (**e**) The detection of MMP-9 cell-surface expression levels by flow cytometry. p, proliferative phase; s, secretory phase. 1, 3, 5 and 7 are the untreated condition; 2, 4, 6 and 8 are the E2 treatment. The histogram represents three independent assays. Data are presented as the mean ± S.E. *, *p* < 0.05 for differences between cell types with the same treatment

To determine whether the OPN and MMP-9 expression levels were influenced by E2, we treated EECs with E2. Using real-time RT-PCR, we found an escalating trend in OPN and MMP-9 expression with increasing doses of E2; the normalized expression levels peaked at 10 nM E2. This trend was observed in both NEECs and EEECs (Fig. 2a). We also conducted a time-course experiment with E2 and found that the OPN and MMP-9 expression levels in NEECs and EEECs increased over time (Fig. 2b). OPN and MMP-9 expression levels reached their maximum 48 h after the beginning of the intervention. Forty-eight hours and 10 nM were the optimum time and concentration for E2 treatment. However, the trends for the dose-dependent and time-dependent reactions from NEECs were weaker than those observed from the EEECs. For NEECs and stage I/II and stage III/IV EEECs, E2 treatment remarkably up-regulated OPN and MMP-9 expression (*p* = 0.018). Significant differences existed between any two groups of NEECs and stage I/II and stage III/IV EEECs after E2 treatment at 10 nM for 48 h (Fig. 2c, *p* = 0.032). For both NEECs and EEECs and in both the proliferative phase and secretory phase, the expression levels of OPN and MMP-9 were significantly different between E2 treatment and the untreated condition (Fig. 2d, *p* = 0.023). Flow cytometry confirmed that the expression of MMP-9 on the surfaces of EEECs was remarkably elevated after 48 h of E2 treatment compared with the NEECs (Fig. 2e). For EEECs, the expression of OPN and MMP-9 in stage III/IV was much higher than that in stage I/II.

### MMP-9 expression reduced upon OPN inhibition by siRNA knockdown

**Fig. 3:**
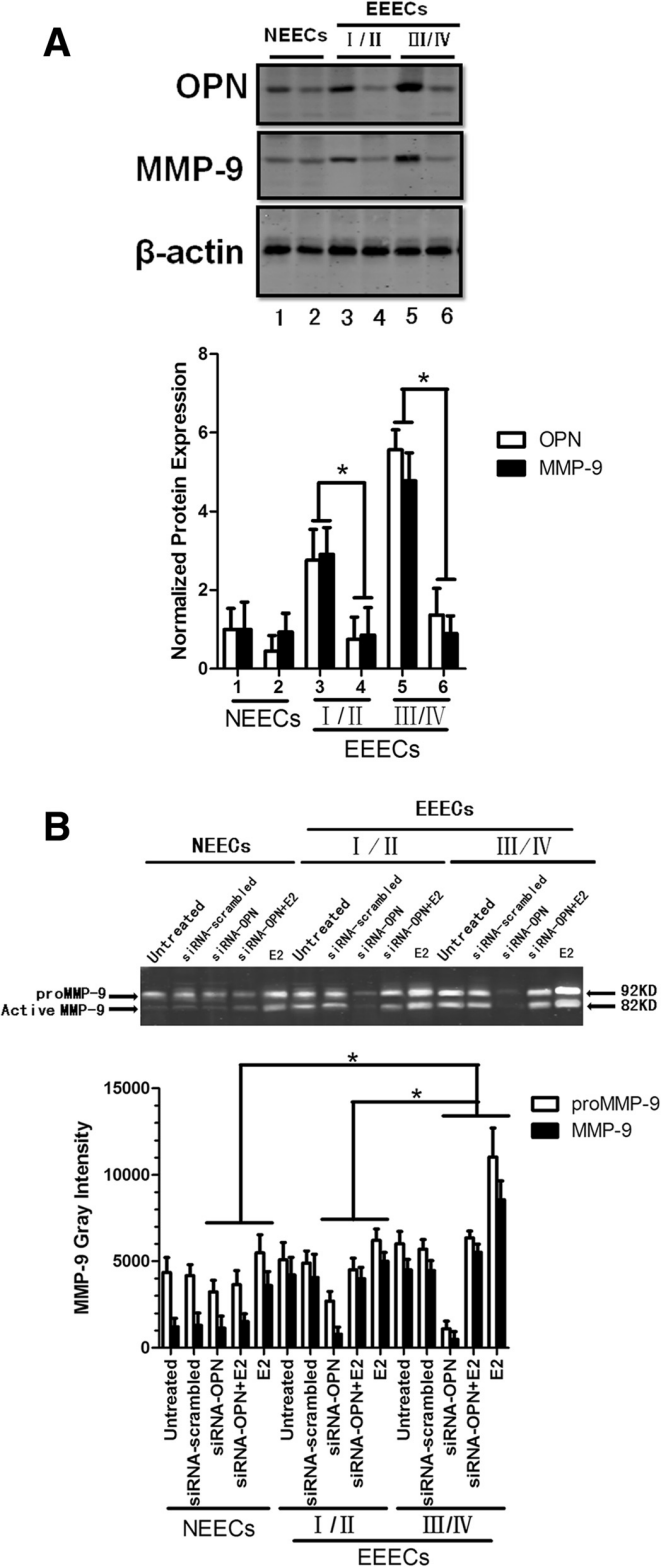
The MMP-9 expression levels and enzymatic activities were detected with western blots and gelatin zymography, respectively, after various treatments. **a** Typical bands from western blots and the histogram are shown for OPN-specific siRNA treatments. 1, 3 and 5 are siRNA-scrambled; 2, 4 and 6 are siRNA-OPN. **b** Representative bands of gelatin zymography and the histogram show the enzymatic activities of MMP-9. The histogram represents three independent assays. Data are presented as the mean ± S.E. *, *p* < 0.05 for differences between cell types with the same treatment

To determine whether MMP-9 expression is regulated by OPN in a signaling pathway similar to that observed in cancer cells, we used siRNA interference to downregulate OPN. OPN expression was specifically downregulated by siRNA-OPN in NEECs and EEECs (Fig. 3a). There was a significant difference in OPN expression levels between the siRNA-OPN and siRNA-scrambled groups in NEECs, stage I/II EEECs and stage III/IV EEECs (*p* = 0.042). Along with OPN, MMP-9 expression was markedly decreased in both stage I/II and stage III/IV EEECs, which was significantly different from the siRNA-scrambled condition (*p* = 0.033). However, after siRNA treatment, MMP-9 expression barely changed in NEECs (*p* = 0.21). To observe the enzymatic activity of MMP-9, we performed gelatin zymography. The conditioned media were collected from NEECs and EEECs. For both cell types, EECs were cultured with siRNA-scrambled, siRNA-OPN, E2, siRNA + E2 or without intervention (untreated). The gray density of gelatin zymography showed that for NEECs, the protein levels of proMMP-9 and MMP-9 hardly changed in any of the intervention conditions (*p* = 0.34). The proMMP-9 and MMP-9 levels from stage I/II and stage III/IV EEECs decreased in the siRNA-OPN condition and increased in the E2 treatment group compared with the untreated group (*p* = 0.017). For the siRNA + OPN groups, the proMMP-9 and MMP-9 levels fell between those of the siRNA-OPN and E2-only groups. We observed that the gray density of EEECs was stronger than that observed in NEECs (Fig. 3b). We also calculated the MMP-9/proMMP-9 ratio and found that the numeric values derived from NEECs were lower than those from EEECs. For the siRNA-OPN, siRNA-OPN + E2 and E2 treatments, there were significant differences between any two groups of NEECs and stage I/II and stage III/IV EEECs (*p* = 0.025).

### E2 and siRNA intervention changed the migratory ability of EEECs

**Fig. 4:**
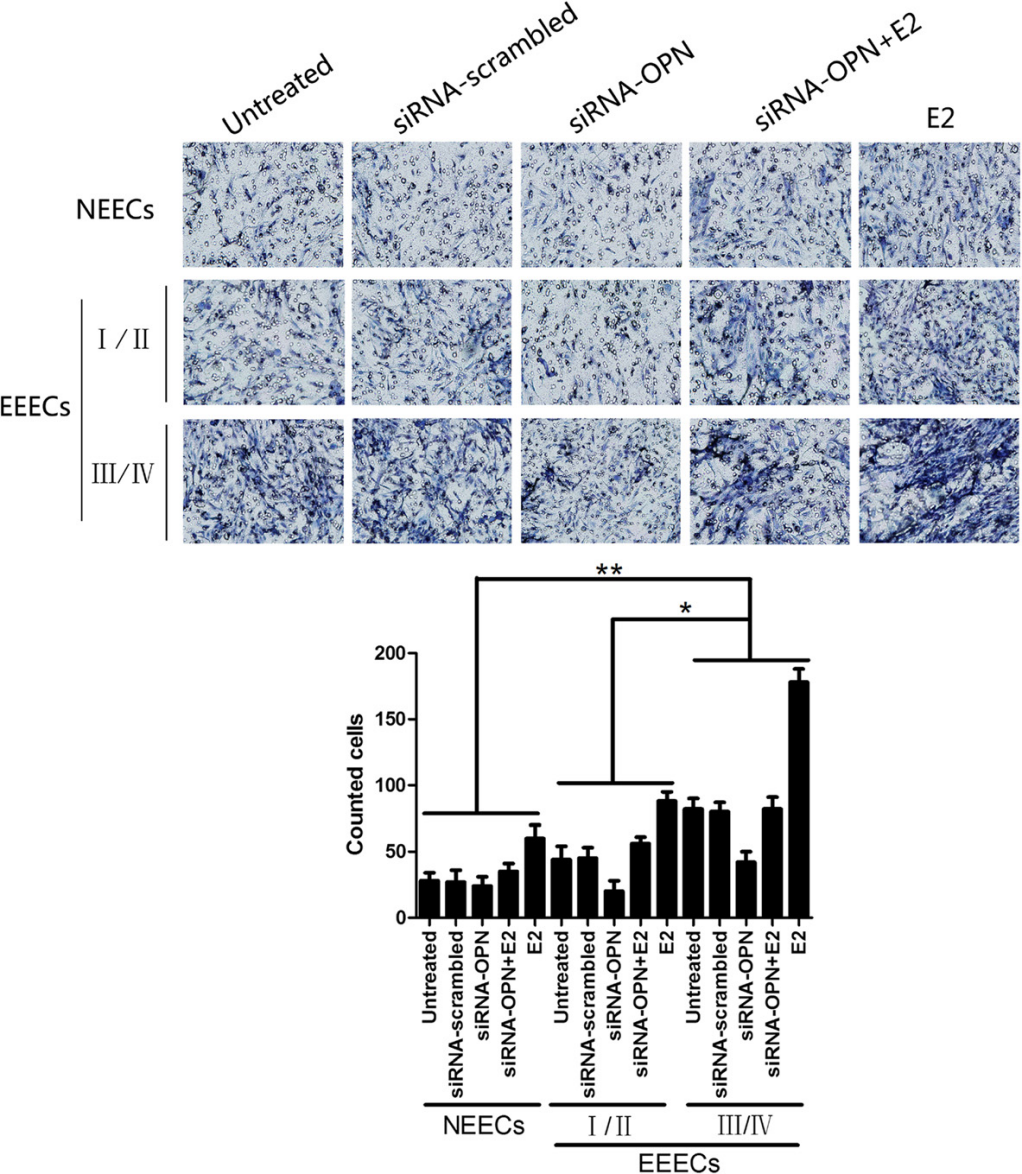
Typical pictures of Transwell assays and the histogram show the altered migration ability of EECs after various interventions. The histogram represents three independent assays. Data are presented as the mean ± S.E. Differences were compared between different cell types with the same treatment, *, *p* < 0.05, **, *p* < 0.01

**Fig. 5:**
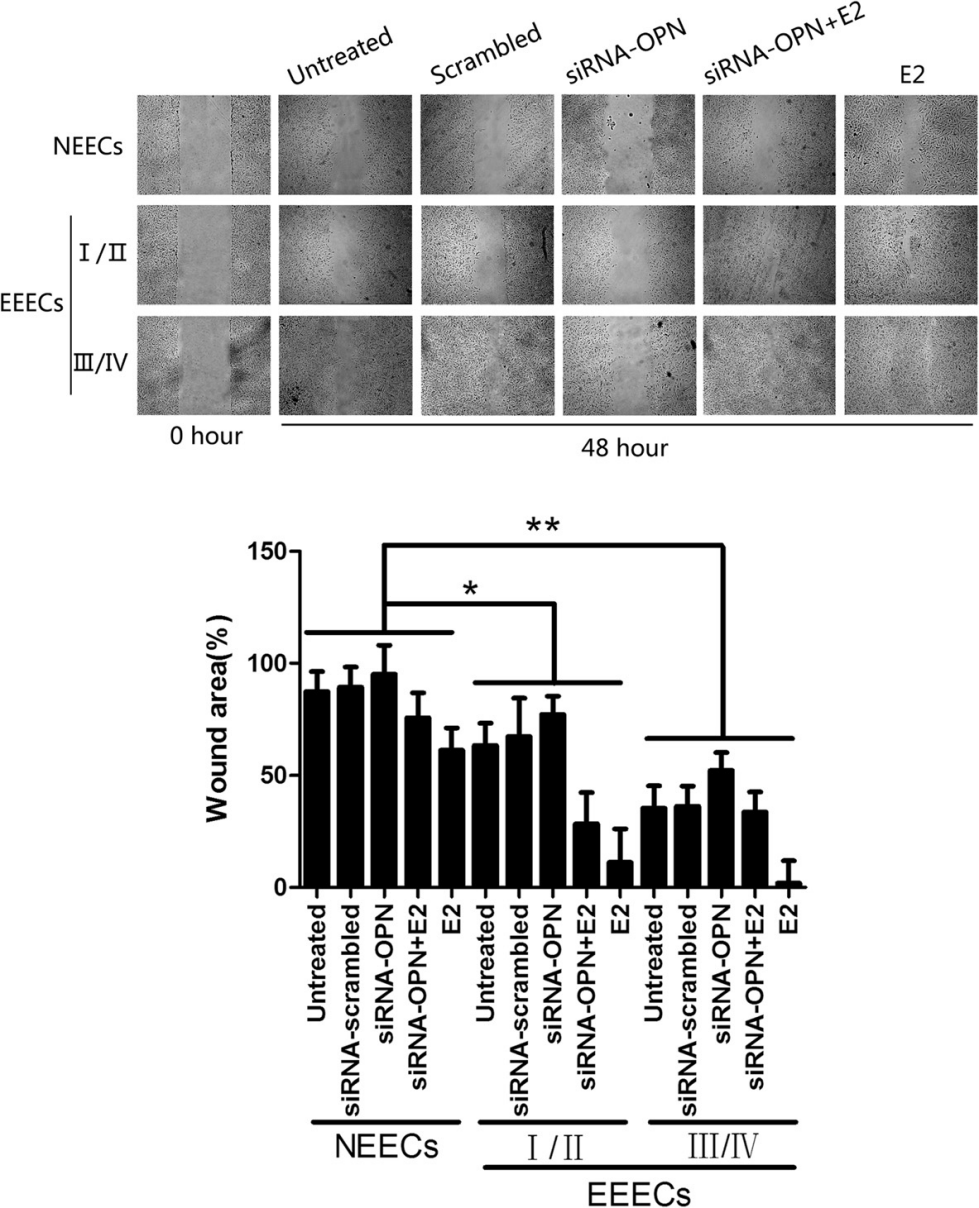
Representative images and the histogram show the results of the wound scratch assay after diverse treatments of NEECs and EEECs. The histogram represents three independent assays. Data are presented as the mean ± S.E. Differences were compared between different cell types with the same treatment, *, *p* < 0.05, **, *p* < 0.01

To observe the differences in the migration abilities of EECs influenced by E2 and siRNA intervention, we performed Transwell and wound scratch assays. For NEECs and EEECs, the numbers of cells counted on the lower surface of the insert membrane of the Transwell for each treatment were E2, siRNA + E2, untreated, siRNA-scrambled and siRNA-OPN in descending order (Fig. 4). E2 was clearly the strongest impetus promoting the migration of EECs, and siRNA-OPN had the strongest ability to inhibit the migration of EECs. Statistical analysis showed that there were significant differences between any two groups of NEECs and stage I/II and stage III/IV EEECs for each of the E2, siRNA + E2 and siRNA-OPN treatments (*p* = 0.040). Similarly, EECs had the strongest ability to migrate after E2 treatment and left the smallest unfilled scratch area. For the siRNA + E2, untreated, siRNA-scrambled and siRNA-OPN treatments, the migratory abilities decreased in wound scratch assays (Fig. 5). Significant differences were observed between any two groups of NEECs and stage I/II and stage III/IV EEECs for the same treatment (*p* = 0.028).

## Discussion

Endometriosis is a common but complex disorder that is believed to be caused by the interaction of various bioactive factors such as steroid hormones, immunological molecules, genetic materials and environmental toxins [1, 26]. According to the theory of retrograde menstruation, endometrial cells can disaffiliate from the normal position and attach to and invade tissues outside the uterus in a manner similar to cancer cell metastasis. That is, a series of actions including disaffiliation, attachment and invasion are of great importance for the development of early endometriosis, and EECs are thought to play an important role in these processes [27]. Many proteins have been reported to correlate with the pathological process of cancers, as well as the pathogenesis of endometriosis [28, 29]. OPN, a protein implicated in the adhesion and migration of cancer cells, was evaluated in normal endometrium and endometrium from individuals with endometriosis. We performed primary EEC culture to exclude the possibility of contamination of endometrial stromal cells. We found that the expression of OPN in EEECs was significantly higher than that in the NEECs. We also found that MMP-9 expression increased in synchrony with increased OPN expression in NEECs and EEECs, which is in agreement with previous reports [11, 14]. For EEECs, the OPN and MMP-9 expression in stage III/IV was higher than in stage I/II. For both NEECs and EEECs, the expression of OPN and MMP-9 was much higher in the secretory phase than in the proliferative phase, which is consistent with previous studies [7, 9]. These results suggest that a cause-and-effect relationship exists between the expression levels of OPN and MMP-9 and that elevated expression of OPN and MMP-9 may initiate the disaffiliation, adhesion and migration of the endometrium to tissues outside the uterus.

To explore whether the elevated expression levels of OPN and MMP-9 in the EEECs are regulated by estrogen, we determined the expression levels of OPN and MMP-9 in NEECs and EEECs after cells were treated with E2. During both the dose–response and the time-course studies, we found that E2 prompted elevated expression of OPN and MMP-9, with a stronger increase observed in EEECs. We also found that after the E2 treatment, the surfaces of EEECs exhibited greater expression of OPN and MMP-9 than the corresponding NEECs. For EEECs, the expression of OPN and MMP-9 on cellular surfaces was much higher in stage III/IV than in stage I/II, which suggested that the OPN and MMP-9 expression levels correlated with the severity of endometriosis or the local E2 concentration. Estrogen is considered a key hormone in the pathogenesis of endometriosis and it exerts its functions by binding to its receptors (mainly ER-alpha and ER-beta). Estrogen receptors have been found to be overexpressed in endometriosis compared with levels in the normal endometrium [30, 31]. Long-term local estrogen accumulation may be responsible for the overexpression of estrogen receptors. Excessive binding of estrogen to receptors activates the expression of downstream genes, many of which play an important role in cell migration. In short, our data support the hypothesis that E2 is a key factor in promoting the overexpression of OPN and MMP-9 in EEECs.

In cancer cells, the OPN/MMP-9 signaling pathway has been reported [5, 6]. To investigate whether a similar pathway exists in EEECs, we performed siRNA interference to specifically down-regulate OPN. Not surprisingly, in EEECs the decreased expression of OPN was accompanied by down-regulated expression of MMP-9. We also treated EEECs with E2 and siRNA-OPN simultaneously. The results show that E2 antagonizes the inhibitory effects of siRNA-OPN on the expressions of OPN and MMP-9. Hence, our data indicate that an MMP-9 signaling pathway regulated by OPN under the local E2 environment exists in EEECs. Interestingly, MMP-9 expression changed only minimally upon OPN down-regulation in NEECs. One possible explanation for this phenomenon is that MMP-9 is regulated by many different signaling pathways, and some signaling pathways are activated during the biological and morphological transformation from NEECs to EEECs. For example, TGF-beta1 and epidermal growth factor can induce EEECs to migrate by selectively activating Raf-1; however, this regulatory pathway has not been reported in NEECs [32]. Combined with the data obtained from the E2 intervention, we infer that OPN may regulate MMP-9 expression in a fluctuating local estrogen environment.

To determine whether there are differences in the amount of proMMP-9 compared with its active counterpart in NEECs and EEECs under various interventions, we performed gelatin zymography. The experimental data showed distinct bands of both proMMP-9 and active MMP-9 in all treatment conditions. The amount of MMP-9 in NEECs was less than that in EEECs. Tissue inhibitors of metalloproteinases (TIMPs), which affect matrix remodeling in normal and pathological conditions, regulate the activity of MMP-9 and maintain the homeostasis in endometrial migration [33]. In fact, the ratios of MMPs and TIMPs have been correlated with the development and progression of endometriosis [34]. TIMPs were reported to have lower expression in endometriosis than in normal conditions. The lower expression of TIMPs in endometriosis means that less MMP-9 is degraded, and EEECs are much more prone to migration. ProMMP-9 is synthesized as a latent, inactive zymogen that can be activated by a number of proteases. In normal and pathological conditions, the expression profiles of proteases are different. New proteases have been found to proteolytically activate proMMP-9 in some cancers but not in the original normal tissues [35]. Our data showed that the ratio of MMP-9/proMMP-9 in NEECs was lower than that in EEECs. These data are in accordance with previous reports [36] and may also be explained by two observations, namely, the reduced MMP-9 degradation and enhanced proMMP-9 activation in EEECs. E2 and OPN are located at specific regulatory sites in the supposed E2/OPN/MMP-9 signaling pathway. Using gelatin zymography, we reconfirmed that MMP-9 is regulated by E2 and OPN via treatment with E2 and OPN-specific siRNA treatment.

To confirm that the migration of EEECs could be affected by the regulation of the E2/OPN/MMP-9 pathway, we performed Transwell and wound scratch assays with different treatments. We found that the number of cells on the lower surface of the membrane and the scratch area left unfilled varied in line with the quantitative changes in OPN and MMP-9 expression caused by the same treatment. These data showed that the E2/OPN/MMP-9 pathway was active in EEECs. We also found that E2 could significantly promote the migration of NEECs, although this effect was inferior to that observed in EEECs. Our data suggest that there may be another signaling pathway that regulates cell migration in NEECs but not in EEECs, which needs to be explored further.

In summary, we performed multiple assays to observe the differential expression of OPN and MMP-9 under various treatment conditions. The aim of these assays was to establish a new signaling pathway in which up-regulation of MMP-9 via activation of OPN induced by estrogen correlates with the migration of endometrial epithelial cells in endometriosis. The basic framework of this signaling pathway has now been established; however, the detailed molecular interactions between E2 and OPN or OPN and MMP-9 are not yet fully elucidated. Additional research will help to elucidate these subjects in the future.

## Abbreviations

BSA: Bovine serum albumin
GnRH-a: Gonadotropin releasing hormone agonists
EEC: Endometrial epithelial cell
GnRH-a: Gonadotropin releasing hormone agonists
NEEC: Normal endometrial epithelial cell
EEEC: Eutopic endometrial epithelial cell
MMP: Matrix metalloproteinase
OPN: Osteopontin
